# 2410. Clinical outcomes and antimicrobial management strategies of *Pseudomonas* endocarditis

**DOI:** 10.1093/ofid/ofad500.2030

**Published:** 2023-11-27

**Authors:** Sunish Shah, Lloyd Clarke, Ryan K Shields

**Affiliations:** Antibiotic Management Program, UPMC Presbyterian Hospital, Pittsburgh, PA, Pittsburgh, Pennsylvania; Antibiotic Management Program, UPMC Presbyterian Hospital, Pittsburgh, PA, Pittsburgh, Pennsylvania; University of Pittsburgh, Pittsburgh, PA

## Abstract

**Background:**

Infective endocarditis secondary to *Pseudomonas* is rare. Our objective was to investigate outcomes of patients with *Pseudomonas* endocarditis by treatment strategy and identify factors associated with treatment failure.

**Methods:**

Adult patients with definitive *Pseudomonas* endocarditis at 8 hospitals were identified between July 2010 and December 2022. Unless the cultured valve yielded a Gram-negative pathogen, patients with blood cultures growing Gram-positive bacteria or yeast for >24 hours were excluded. Combination therapy was defined as receipt of ≥2 in vitro active agents for ≥72 hours. Failure was a defined as death or microbiologic failure by day 42; microbiologic failures included escalation of antimicrobial therapy following treatment-emergent resistance, increased vegetation size, or blood cultures positive for ≥14 days.

**Results:**

34 patients met the inclusion criteria. 32% (11/34) were people who inject drugs and 12% (4/34) were organ transplant recipients. *P. aeruginosa* was the underlying pathogen in 97% (33/34) of patients. 65% (22/34) and 35% (12/34) received combination and monotherapy, respectively. Patients managed with combination therapy had comparable rates of prosthetic valve endocarditis (41% vs 17%, P=0.25), a vegetation size > 1cm (59% vs 42%, P=0.33) and no receipt of cardiac surgical intervention despite an indication (32% vs 17%, P=0.44) compared to those who received monotherapy. There was no significant difference in 42-day failure rates (32% vs 17%, P=0.44), 90-day mortality (18% vs 25%, P=0.68) or median hospital length of stay (22 days vs. 23, P=0.914) between patients who receivedcombination therapy and monotherapy. Patients who experienced 42-day failure were more likely to have multi-drug resistant (MDR) *Pseudomonas* on initial blood cultures (44% vs 4%, P=0.012) and were less likely to be initially managed with piperacillin/tazobactam or cefepime (44% vs 96%, P=0.004) compared to those who experienced clinical cure.

Combination therapy versus monotherapy

**Figure:**
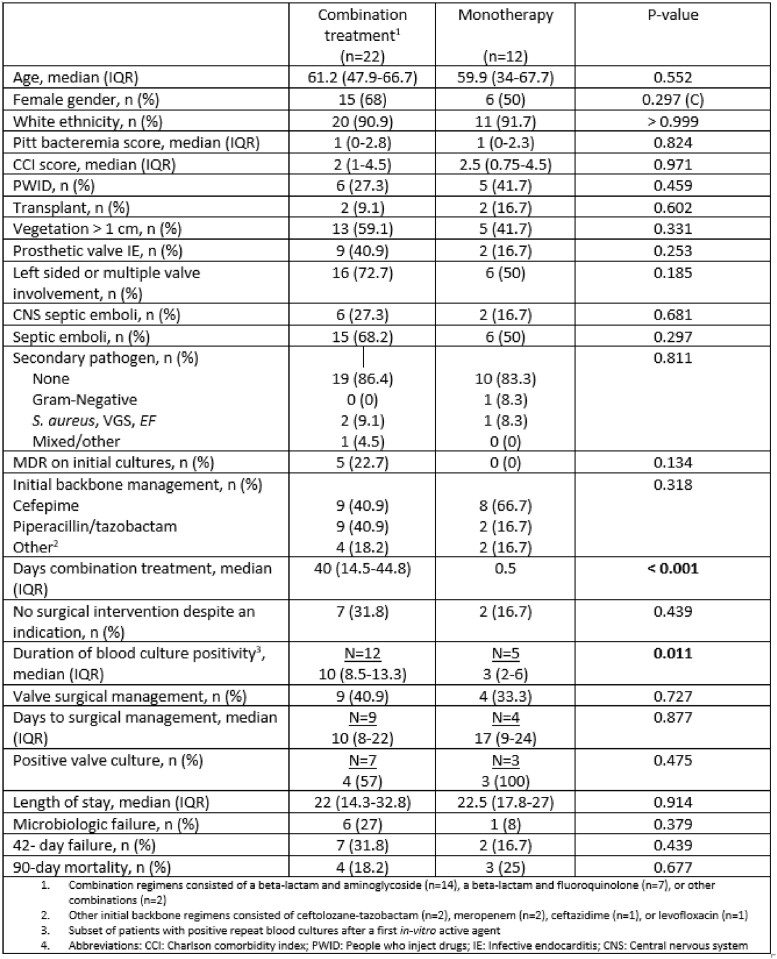

Factors associated with clinical failure

**Figure:**
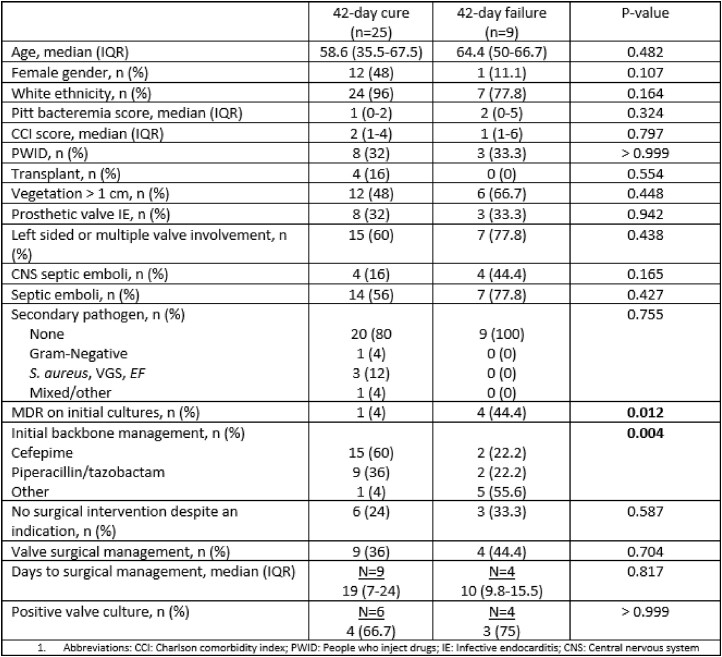

**Conclusion:**

To our knowledge, this is the largest study of *Pseudomona*s endocarditis to date. We did Not identify a benefit to combination therapy; however, the overall sample size was small. Endocarditis due to MDR *P. aeruginosa* was associated with a higher risk of clinical failure.

**Disclosures:**

**Ryan K. Shields, PharmD, MS**, Allergan: Advisor/Consultant|Cidara: Advisor/Consultant|Entasis: Advisor/Consultant|GSK: Advisor/Consultant|Melinta: Advisor/Consultant|Melinta: Grant/Research Support|Menarini: Advisor/Consultant|Merck: Advisor/Consultant|Merck: Grant/Research Support|Pfizer: Advisor/Consultant|Roche: Grant/Research Support|Shionogi: Advisor/Consultant|Shionogi: Grant/Research Support|Utility: Advisor/Consultant|Venatorx: Advisor/Consultant|Venatorx: Grant/Research Support

